# Tumor Associated Macrophages: Origin, Recruitment, Phenotypic Diversity, and Targeting

**DOI:** 10.3389/fonc.2021.788365

**Published:** 2021-12-20

**Authors:** Tetiana Hourani, James A. Holden, Wenyi Li, Jason C. Lenzo, Sara Hadjigol, Neil M. O’Brien-Simpson

**Affiliations:** Antimicrobial, Cancer Therapeutics and Vaccines (ACTV) Research Group, Melbourne Dental School, Centre for Oral Health Research, Royal Dental Hospital, The University of Melbourne, Melbourne, VIC, Australia

**Keywords:** tumor associated macrophages (TAMS), solid tumor, peptide, nanotargeting, nanotherapy, cancers, immunotherapies

## Abstract

The tumor microenvironment (TME) is known to have a strong influence on tumorigenesis, with various components being involved in tumor suppression and tumor growth. A protumorigenic TME is characterized by an increased infiltration of tumor associated macrophages (TAMs), where their presence is strongly associated with tumor progression, therapy resistance, and poor survival rates. This association between the increased TAMs and poor therapeutic outcomes are stemming an increasing interest in investigating TAMs as a potential therapeutic target in cancer treatment. Prominent mechanisms in targeting TAMs include: blocking recruitment, stimulating repolarization, and depletion methods. For enhancing targeting specificity multiple nanomaterials are currently being explored for the precise delivery of chemotherapeutic cargo, including the conjugation with TAM-targeting peptides. In this paper, we provide a focused literature review of macrophage biology in relation to their role in tumorigenesis. First, we discuss the origin, recruitment mechanisms, and phenotypic diversity of TAMs based on recent investigations in the literature. Then the paper provides a detailed review on the current methods of targeting TAMs, including the use of nanomaterials as novel cancer therapeutics.

## Introduction

The tumor microenvironment (TME) that surrounds cancer cells, plays an important role in carcinogenesis. It is involved in multiple processes including tumor growth, metastasis, and the development of treatment resistance (1). The TME is known to be unique to each tumor type and changes throughout the different stages of tumor development, thus making TME biology a complex field of research. The TME consists of various components, such as tumor stromal cells (cancer associated fibroblasts), blood and lymphatic vasculature, immune cells (macrophages, tumor infiltrating lymphocytes, dendritic cells, etc), and non-cellular components such as extracellular matrix and signalling molecules. The continual interaction of these components along with malignant cells provides a dynamic network for either supporting or suppressing tumorigenesis (2, 3).

**Figure 1:**
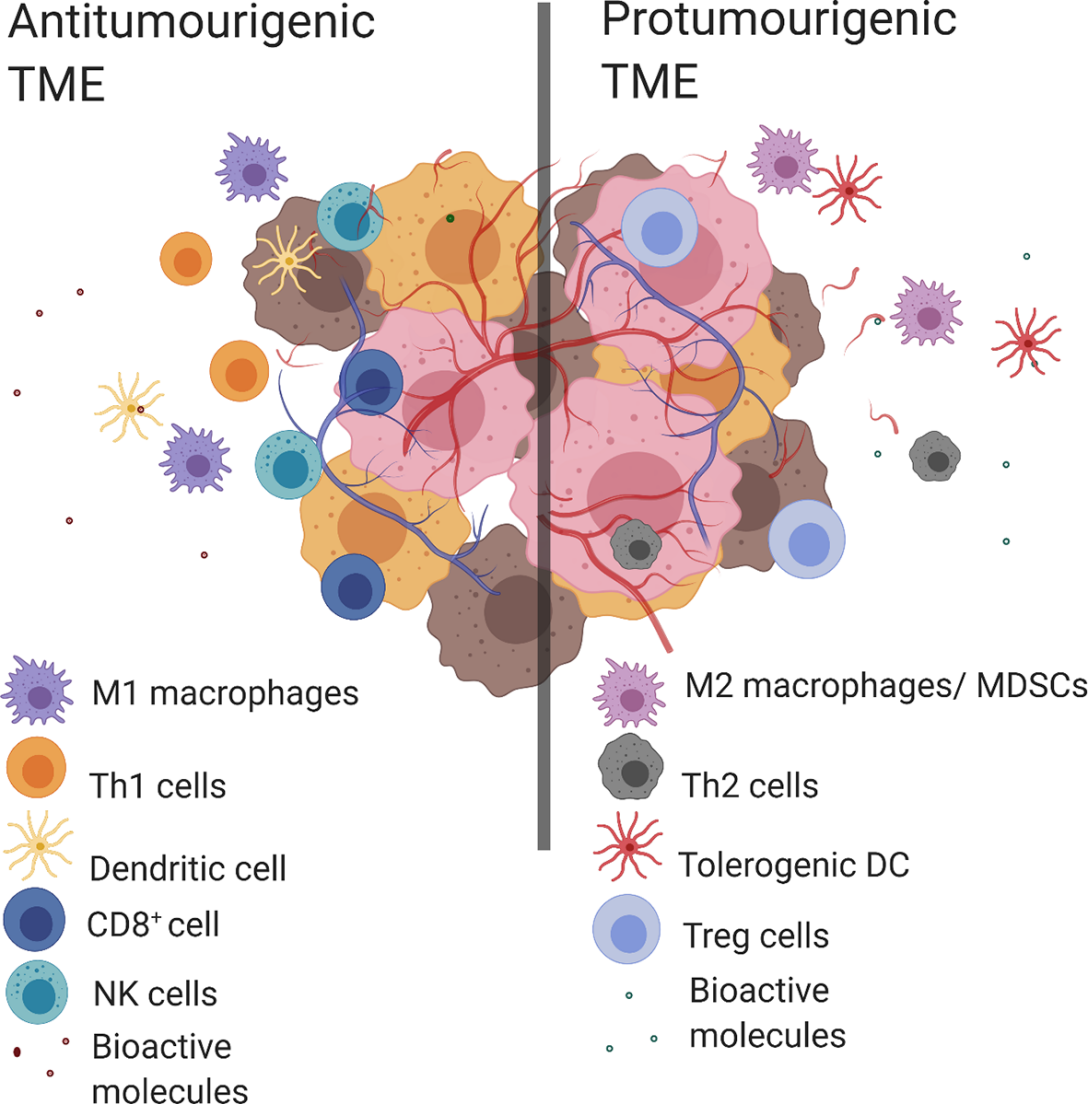
Antitumorigenic and protumorigenic TME components. Antitumorigenic and protumorigenic TME have distinct immunologic profiles. Left. An antitumorigenic TME: M1 macrophages, Th1 cells, DC, CD8+ cells, NK cells and bioactive molecules (antitumorigenic cytokines (IL-2, IL-12, IFNγ), growth factor (GM-CSF), chemokines (CXCL9, CXCL10). Right. A protumorigenic TME: M2 macrophages, tolerogenic DCs, MDSCs, Th2 cells, Treg cells and bioactive molecules (protumorigenic cytokines (IL-4, IL-6, IL-10, TGF-β, IFNγ), angiogenic factors (VEGF), growth factors (GM-CSF, EGF, HGF, FGF) and chemokines (CCL2)). Created with BioRender.com.

Tumor evasion of the immune system is a known “hallmark of cancer” where clinical outcomes have been consistently linked to immune cell populations present within the TME (2). An antitumorigenic TME is characterized by the presence of multiple types of immune cells including T helper cells of type 1 (Th1), CD8+ cytotoxic T lymphocytes (CTL), M1 macrophages, N1 neutrophils, natural killer cells (NK cells) and dendritic cells (DC). This coincides with proinflammatory bioactive molecules such as; antitumorigenic cytokines [interleukin 2 (IL-2), IL-12, interferon gamma (IFNγ)], growth factors [granulocyte-macrophage colony-stimulating factor (GM-CSF) and chemokines (CXC motive chemokine ligand 9 (CXCL9), CXCL10]. Conversely, the predominant immune cell populations in a protumorigenic TME include: M2-like TAMs, T helper 2 cells (Th2), myeloid-derived suppressor cells (MDSC), N2 neutrophils, tolerogenic dendritic cells (tDC), and T regulatory cells (Tregs). While the protumorigenic bioactive molecules include; protumorigenic cytokines (IL-4, IL-6, IL-10, transforming growth factor beta (TGF-β), IFNγ), angiogenic factors (vascular endothelial growth factor (VEGF), growth factors [GM-CSF, epidermal growth factor (EGF), hepatocyte growth factor (HGF), fibroblast growth factor (FGF)] and chemokines [C–C motif chemokine ligand 2 (CCL2)] (2, 4) (**Figure 1**). The complex structure of the TME means that the presence of certain cell populations and/or biological molecules are not always indicative of tumor progression or suppression (5–11). For example, IFNγ is predominantly known for its antitumor activity, however it has been shown to have a protumorigenic property by inducing the upregulation of PD-L1 in tumors (12–14). Another example is GM-CSF which is recognized for its ability to induce antitumor immune responses as well as modulate protumorigenic properties like tumor growth and spread (15).

With the increased interest in the protumorigenic immune TME, more opportunities arise in discovering novel therapeutic targets and in developing potent anticancer therapies. For instance, the recent introduction of immune checkpoint inhibitors (ICI) improved treatment outcomes for many cancer patients and highlighted the importance of the research in this field (16, 17). Unfortunately, many cancers become or are non-responsive to ICI and investigations are ongoing to understand underlying cause of resistance (18). Currently, a number of clinical trials are investigating the therapeutic potential of combining ICI with targeting TAMs in order to counteract a protumorigenic TME and overcome ICI resistance (19).

Multiple studies describe the active role of TAMs in tumorigenesis and demonstrate that targeting protumorigenic TAMs is an effective strategy to attenuate tumor progression (20–23). The aim of this paper is to provide a current understanding of macrophage biology in relation to their role in tumor progression, and therapeutic approaches to target TAMs, including a specific targeting with nanomaterials and TAM targeting peptides.

## Macrophage Heterogeneity, Plasticity, and Nomenclature

Macrophages are a heterogeneous and complex population of immune cells having roles in host defense against pathogens, maintenance of tissue homeostasis and tissue architecture (24). Macrophages are highly plastic cells and in response to microenvironmental signals such as chemokines and cytokines differentiate/polarize into distinct phenotypes with specific functionality. Multiple populations of macrophages are known to be present within the same microenvironment and each phenotype has a distinctive combination of expressing receptors, secreting chemokines and cytokines (25). A current classification of macrophages is based on their function and response to polarizing agent. Traditionally, unpolarized macrophages are called M0 (naïve) and all other macrophage phenotypes have been divided between M1 (classical) and M2 (non-classical) polarization spectra. This classification also includes M2 subtypes M2a, M2b, M2c, M2d (26) (**Table 1**). Since the same/similar phenotypes can be generated by several polarizing agents *in vitro*, Murray et al. suggested to expand macrophage classification by including the activating molecules in macrophage naming. According to this nomenclature, M1 macrophages are subdivided into M(LPS), M(IFNγ) or M(IFNγ+LPS), M2a become M(IL-4), M(IL-13) or M(IL-4/IL-13), M2b – M(IC+LPS), M2c – M(IL-10) etc (38). Mosser et al. have also proposed to categorize macrophages accordingly to their *in vivo* functions, such as: (i) host defense, performed by classically activated macrophages induced by IFNγ, LPS, and TNF, (ii) wound healing, performed by IL-4 and IL-13 activated macrophages, and (iii) immune regulation, performed by regulatory macrophages induced by glucocorticoids, TGF-β, IC+LPS, IL-10, apoptotic cells, prostaglandins, adenosine and some other stimulants (39). Though the M1-M2 classification system is used extensively, its appropriateness and *in vivo* applicability are debated, as there are macrophage subsets that express an intermediate phenotype and have markers present from both M1 and M2 polarization states (24). Additionally, there are macrophage phenotypes with novel characteristics (40, 41). Current theorems suggest that the development of an immunologically relevant macrophage classification also depends on an understanding of the macrophage’s surrounding microenvironment (24, 38, 40). Indeed, with increased investigations into macrophage polarizing agents, more novel macrophage phenotypes are being discovered. For example, two recently identified macrophages, M4 and M17, are defined by their polarizing ligands chemokine (C-X-C) ligand 4 (CXCL4) (40) and IL-17 (42), respectively (**Table 1**). In addition, novel macrophage phenotypes have been discovered by a transcriptome analysis of human macrophages polarized with 28 different stimuli. Interestingly, some of these transcriptomes were matched to the transcriptomes of alveolar macrophages obtained from smokers and patients with chronic obstructive pulmonary disease (COPD) (41).

**Table 1:** *In vitro* macrophage phenotypes and their role in cancer.

Phenotype	Activators	Phenotypic Markers/Secreted molecules	Association with cancer
M1 (classical)	IFNγ/Lps TNF	CD86, iNOS, CD80, CD40, CD69, MHCII, CD38, TLR2, TLR4; IL-12, IL-23, IL-6;	Improved patient survival in NSCLC (27) and ovarian cancer (28).
M2a (M2, alternative)	IL-4/IL-13	CD206, CD163, MHCII, Erg2; TGF‐β, IL-10, IL-1RA; In mouse only: Arg-1, Ym1/2, Fizz1(RELM- α);	Lung cancer progression (29). Stimulated invasion and migration of breast cancer cells (30).
M2b (regulatory)	Immune complexes/LPS, IL-1R	CD86, CD163, MHCII, iNOS; LIGHT (TNFSF14), CCL1, IL-10, IL-1, IL-6, TNF- α;	Progression of HCC (31). Bevacizumab-resistant triple-negative breast cancer (32).
M2c (regulatory)	IL-10, Glucocorticoids, TGF- β	CD163, TLR1, TLR8, CCR2, SR-A (CD204); IL-10, TGF- β;	Progression of lung cancer (29). Advanced breast cancer (33).
M2d (angiogenic, TAMs)	Adenosine/Lps, IL-6, LIF	iNOS, IL-10, IL-12, IL-6, VEGF;	Tumor angiogenesis (34) Progression of gastric cancer (35) and HCC (36). Radiation-induced macrophage infiltration in NSCLC (37).
M4	CXCL4	IL-6, TNF, MMP-7, MMP-12;	Not reported
M17	IL-17	TLR2, TLR4; TNF- α;	Not reported

Overall, traditionally M1 and M2 polarization states are used to classify macrophages. However, with advancements in medical research it has become evident that *in vivo* macrophage diversity is more complex. With the increased understanding of macrophage polarizing conditions, more novel macrophage phenotypes are being discovered that not only demonstrate macrophage heterogeneity and plasticity but are also more suitable *in vitro* models.

### TAMs Origin and Recruitment

Macrophages have two distinct developmental pathways. Briefly, tissue-resident macrophages are developed from embryonic precursors (fetal yolk sack or fetal liver progenitors) and monocyte-derived macrophages are developed from bone-marrow haemopoietic cell progenitors (43). Tissue-resident macrophages are widely distributed in the body and, depending on their location, are called osteoclasts in bone, alveolar macrophages in lungs, microglial cells in central nervous system and Kupffer cells in liver. Monocyte-derived macrophages serve as a reservoir for macrophage replenishment and are recruited in pathology (25). Under physiological conditions, tissue resident macrophages and monocyte-derived macrophages have a distinct tissue distribution: (i) both subsets are present in liver, pancreas, lung, heart, kidney and spleen; (ii) only tissue-resident macrophages (yolk sac derived) are found in brain; (iii) monocyte-derived macrophages predominate in intestines and dermis (44, 45).

**Figure 2:**
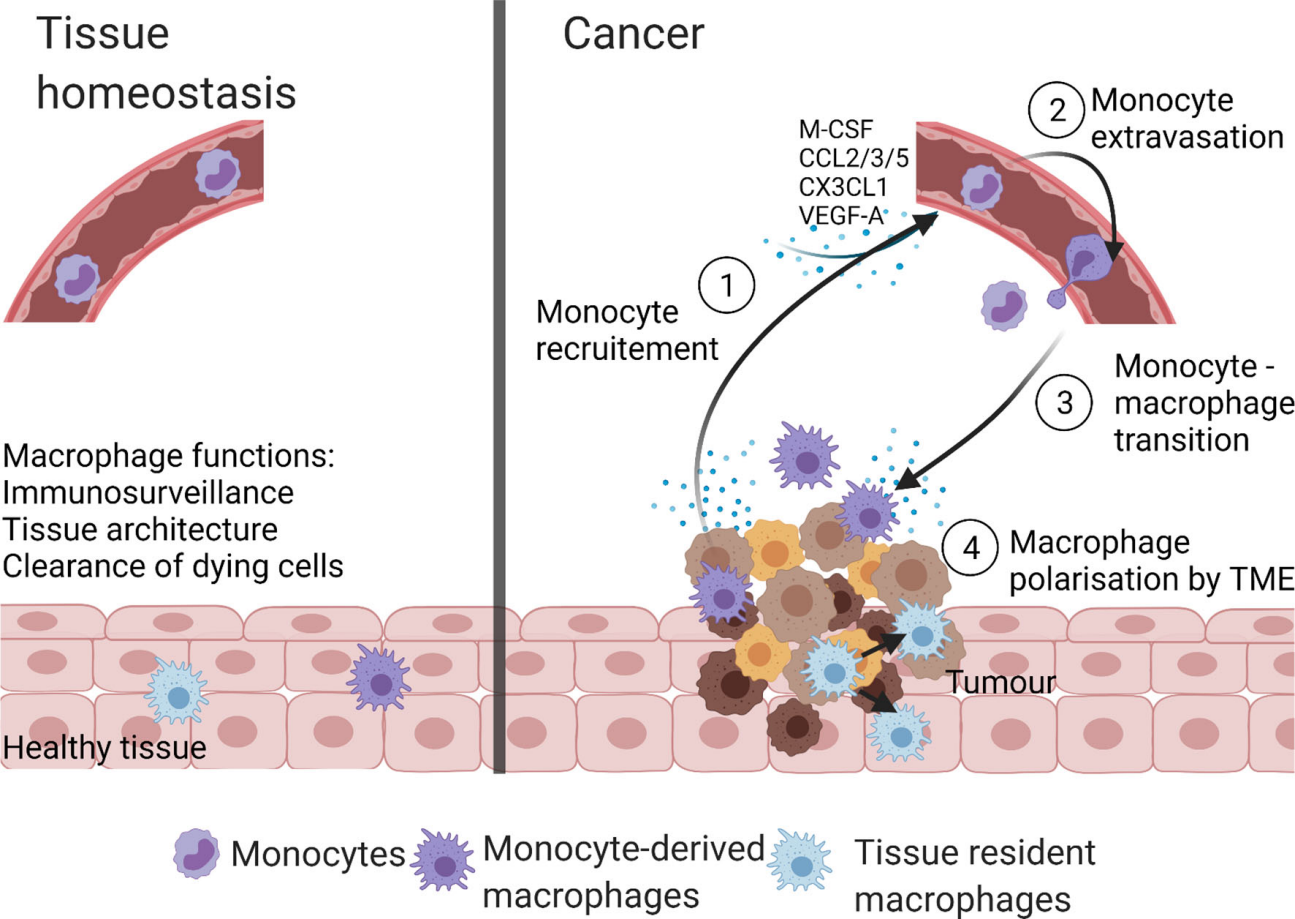
Macrophage populations in homeostasis and cancer. In health, macrophages present within tissues are tissue-resident macrophages and/or monocyte-derived macrophages. These macrophages maintain tissue homeostasis by performing numerous functions like immunosurveillance, clearance of senescent and apoptotic cells, and maintenance of tissue architecture. The TME of cancer is highly infiltrated with macrophages due to the increase in monocyte recruitment from the blood stream and/or self-proliferation of tissue-resident macrophages. The TME derived monocyte recruiting molecules (M-CSF, CCL2, CX3CL1, CCL3, CCL5, and VEGF-A) stimulate monocyte extravasation into the TME, where monocytes are transformed to monocyte-derived TAMs. Created with BioRender.com.

Compared to tissue homeostasis, cancer is characterized by increased monocyte recruitment and/or expansion of tissue-resident macrophages with both populations involved in tumorigenesis (25) (**Figure 2**). For example, in a murine model of pancreatic ductal adenocarcinoma (PDAC), the expansion of the tissue resident-macrophages population (>29 fold) was observed (44). Both, tissue resident interstitial macrophages and recruited monocyte-derived macrophages were found in murine TC-1 lung carcinoma (46). Chen et al. reported that recruited monocyte-derived macrophages accounted for 85% of the total TAMs in glioblastoma (47). Recruitment of monocyte-derived macrophages to liver was also seen in hepatocellular carcinoma (48).

Various pathways are involved in monocyte recruitment to the tumor including monocyte recruiting cytokines, chemokines, and growth factors. The most prominent recruitment ligand/receptors are M-CSF/CSF-1R, CCL2/CCR2, CX3CL1/CX3CR1, CCL3/CCR1, CCL3/CCR5, CCL5/CCR5, and VEGF-A/VEGFR1 ligand-receptor interactions (49).

M-CSF (also called CSF-1) is an important molecule involved in recruitment and M2 polarization of monocytes, and in self-renewal of tissue-resident macrophages (50, 51). High levels of expression of M-CSF was found in advanced upper tract urothelial carcinoma (52), breast cancer (53), esophagus SCC (54), gastric cancer (55).

Colony-stimulating factor 1 receptor (CSF-1R) has two ligands, macrophage colony-stimulating factor (M-CSF) and IL-34, and both are involved in various protumorigenic processes. IL-34 has two effects on tumor cells and the TME, being protumorigenic in lung cancer and ovarian cancer, but having an antitumorigenic role in luminal and Her2+ breast tumors. The protumorigenic effects of IL-34 on TAMs include monocyte recruitment, polarization, and survival in the TME (56).

The chemokine CCL2 is a strong chemoattractant for CCR2+ monocytes (57) and its overexpression was reported in progressing breast cancer (58), prostate cancer (59), oral squamous cell carcinoma (OSCC) (60), liver cancer (61), and colorectal cancer (CRC) (62). A number of studies have shown a correlation between CCL2 expression and TAM infiltration in clear cell renal cell carcinoma (63) and esophageal cancer (64). CCL8, another ligand of CCR2, was found to be highly overexpressed in human cervical cancer and involved in TAMs recruitment to hypoxic areas (65).

Another chemokine involved in monocyte recruitment is CCL3. Interestingly, CCL3 has diverse functions in the TME as it is secreted by a broad range of immune/non-immune cells and, in addition, CCL3 binds to various cells expressing CCR1, CCR5, and CCR3 (5, 66). Increased levels of CCL3 have been reported in colorectal (67), esophageal (68), and endometrial cancers (5). CCL3 secretion by TAMs was found to be induced by CCL2/CCR2 and IL-33, with these TAMs reported to mediate metastatic spread in different tumor models (66, 68, 69). For example, the CCL3/CCR1 axis was found to mediate macrophage recruitment during the formation of early metastatic niches in a mouse E0771-LG breast cancer model (66, 69), and CCL3/CCR5 mediated macrophage recruitment to the metastatic site in murine Renca renal cell carcinoma model (70). In addition, CCL3 derived from TAMs was reported to promote esophageal cancer cells (TE-8 and TE-9) migration and invasion *via* interaction with their CCR5 receptor (68).

CCL5 is also a known potent monocyte chemoattractant. Similar to CCL3, CCL5 is expressed by a variety of cell types, and has a number of binding receptors including CCR1, CCR3, CCR5, G-protein coupled receptor 75 (GPR75), and CD44 (5, 71). High expression of CCL5 has been correlated with poor prognosis in many cancers (5), including glioblastoma (71) and breast cancer (72). Indeed, CCL5 was shown to recruit and mediate M2-like TAM polarization, with this being linked to glioblastoma progression (71), as well as to recurrence and metastasis in breast cancer (73, 74). Apart from monocyte recruitment, CCL5 has a direct effect on tumor cells, supporting tumor cells migration, invasion, and survival (71, 74). High CCL5 expression was also associated with increased TILs such as CD8+ T cells, NK cells, and M1 macrophages in triple negative breast cancer. This study reported that patients with low CCL5 and TILs had an increased residual tumor size (75).

The CX3CL1/CX3CR1 axis is also an important monocyte recruitment pathway. In human colon carcinoma, CX3CR1+ TAM infiltration was associated with poor prognosis as these TAMs were found to be proangiogenic and prometastatic (76). The CX3CL1/CX3CR1 axis was also involved in macrophage recruitment in breast cancer (77). In addition, CX3CR1+ and CCR2+ recruited macrophages were also found to support Lewis lung carcinoma progression (78). While predominantly CX3CL1/CX3CR1 signalling is related to tumor progression, inactivation of the CX3CL1/CX3CR1 axis was shown to enhance glioblastoma development. In fact, brain tissue homeostasis is maintained by CX3CR1+ microglia which interact with CX3CL1+ neurons (79).

TAM recruitment to the hypoxic TME is also mediated by VEGF-A, endothelin-2, and EMAPII. Once recruited, TAM migratory receptors become downregulated, which prevents their further migration. Within a hypoxic TME, TAMs participate in angiogenesis and tumor spread due to the upregulation of hypoxia-inducible factor (HIF)-1α and HIF-2α (80). HIF-1α is known to increase TAMs infiltration and induces epidermal growth factor (EGF) production in TAMs, which in turn promotes metastasis (81). Further, both HIF-1α and HIF-2α upregulate angiogenic molecules VEGF, IL-6, and the tyrosine-protein kinase receptor (Tie2) receptor in TAMs, thus promoting tumor angiogenesis (81, 82).

It is clear that tissue-resident-macrophage expansion and new monocyte recruitment are critical for the development of multiple solid cancers. Indeed, these studies suggest that the major monocyte recruiting pathways (M-CSF/CSF-1R, CCL2/CCR2, CCL3/CCR1, CCL3/CCR5, CCL5/CCR5 and CX3CL1/CX3CR1) enable tumor survival by supplying the TME with pro-tumorigenic TAMs. Thus, a suppression of monocyte/macrophages recruitment is a potential therapeutic strategy to eliminate or reduce TAMs involvement in tumorigenesis.

### Protumorigenic Tumor Associated Macrophages (TAMs)

**Figure 3:**
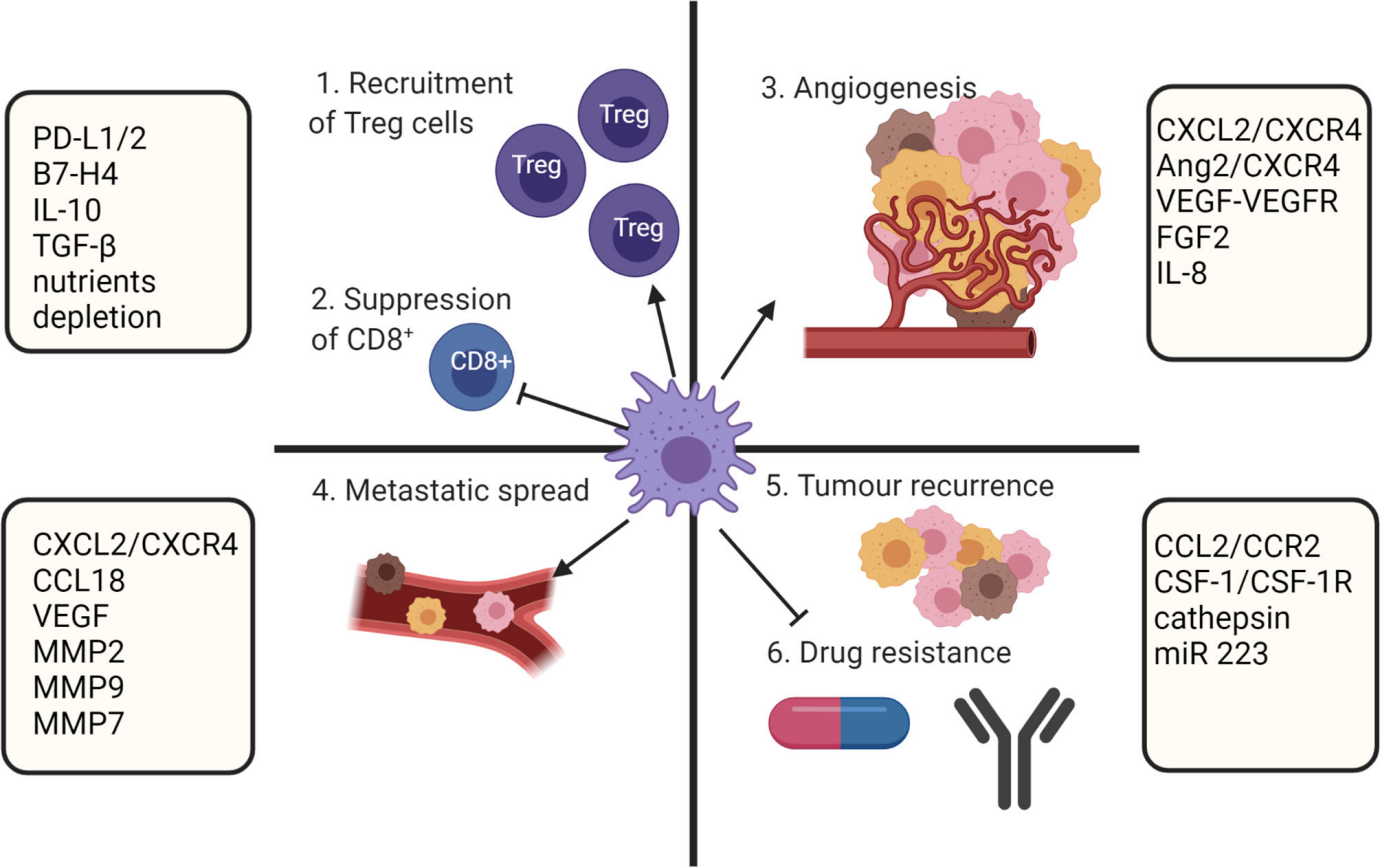
TAMs role in supporting tumor growth. TAMs mediate immunosuppression by recruiting Treg cell and inhibiting CD8+ T cells. In addition, TAMs participate in metastatic spread, angiogenesis, and drug resistance. Created with BioRender.com.

Biomolecules derived from the TME and cancer cells have a direct effect on macrophage polarization. Although at the early stages of tumor formation macrophages within the TME predominantly express a proinflammatory M1 phenotype, tumor progression is associated with increased infiltration of anti-inflammatory M2 polarized TAMs (83). It is known that the TAM population within the TME is phenotypically heterogenous (25, 84) and the overall number of TAMs accumulated within a tumor is not considered in estimation of clinical prognosis. However, the ratio of M1/M2 is considered an important prognostic marker (6–11). A low M1/M2 TAM ratio is associated with tumor progression and poor prognosis, while a high M1/M2 ratio tends to correlate with positive outcomes in ovarian cancer (6), gastric cancer (7), CRC (8), osteosarcoma (9), lung cancer (10) and OSCC (11). Multiple studies showed that M2 TAMs have strong roles in promoting of tumor growth (85), angiogenesis (86, 87), modification of extracellular matrix (88), inhibition of anti-tumor immunity (25), metastasis (89), chemoresistance (90) and recurrence (91) (**Figure 3**).

TAMs mediated immunosuppression severely disrupt the effector cell functions required for tumor clearance. Some of the known strategies by which TAM mediate immune evasion include: (i) the upregulation of checkpoint inhibitors such as programmed cell death ligand 1 (PD-L1), PD-L2 and B7 superfamily member 1 (B7-H4) which deactivates effector CTLs *via* interaction with their receptor PD-1. Increased expression of PD-L1 and PD-L2 within the TME has been associated with poor prognosis in multiple solid cancers (92) and CTL dysfunction was mediated by B7-H4 expressing TAMs in ovarian cancer (93); (ii) production of immunosuppressive cytokines IL-10 and TGF-β that affect multiple immune cells. Both cytokines are known to induce an anti-inflammatory phenotype in monocytes and macrophages and inhibit CD4+ and CD8+ T cell proliferation and cytokine production (94, 95). In addition, TGF-β induces Treg cells and prevents Th1 polarization (95). Interestingly, TAM derived IFNγ has been shown to induce PD-L1 expression in lung cancer and by this mechanism supported tumor progression (96); and (iii) CTL exclusion from intra-tumoral environment. The known TAMs mechanisms are stimulation of tumor stroma fibrosis by granulin secretion and depletion of nutrients (97). Overall, TAMs are equipped with multiple mechanisms to generate and support an immunosuppressive TME.

TAMs are known to be strongly involved in tumor angiogenesis and it has been reported that the hypoxic environment of the TME aids this TAM function. Angiogenic TAMs are typically located in hypoxic avascular tumor areas (98) with recruitment and maintenance of angiogenic function mediated by CXCL12-CXCR4, angiopoietin-2 (Ang2)-Tie2, and VEGF-VEGFR pathways (87). Under hypoxic conditions TAMs are known to upregulate HIF-1α/2α (80) which are inducers for angiogenic molecules like VEGF, FGF2, CXCL8, IL-8, and the Tie2 receptor (80–82).

Some TAM phenotypes have a distinct role in tumor metastasis. For example, CXCR4+ TAMs are involved in metastatic spread *via* promotion of epithelial-to-mesenchymal transition (EMT) and cancer stem cells development in OSCC (99), luminal B breast cancer (100) colorectal cancer (101), non-small cell lung cancer (NSCLC) (102), hepatobiliary cancers (103). These TAMs are recruited to the tumor site by stromal cell derived factor 1 (CXCL12), a chemokine produced within the TME (104). Metastasis was also supported by CCL18 secreting TAMs in pancreatic ductal adenocarcinoma (PDAC) (105), head and neck squamous cell carcinoma (HNSCC) (106), osteosarcoma (107), gallbladder cancer (108), hepatocellular carcinoma (HCC) (109), NSCLC (110), gastric cancer (111). CCL18 is a chemokine involved in immune tolerance by the recruitment and generation of immune cells like tolerogenic DCs, Th2, M2 macrophages and suppression of effector T lymphocytes. In addition, exposure of tumor cells to CCL18 promoted EMT cell motility and invasion (112). TAMs are known to produce proteolytic enzymes like matrix metalloproteinases (MMP2, MMP9, MMP7), serine proteases, and factors involved neovasculature formation (VEGF, MMP9) to enhance metastasis (113). As such, TAM derived MMP9 has been associated with enhanced invasion and migration in HCC (114), gastric cancer (115), melanoma (116), colon cancer (117), and estrogen receptor positive breast cancer (118).

Tumor recurrence has been shown to be associated with increased TAM infiltration in the TME. For example, increased CD11b+ myeloid cells infiltration was observed in early recurrent head and neck cancer (119) and in recurrent glioblastoma (120). In addition, reduced recurrence-free survival was associated with increased stromal CD68+ CD163+ TAMs in basal-like breast cancer (121), and with increased tumor center CD68+ CD163+TAMs in colorectal cancer (122).

TAM mediated chemoresistance is a significant challenge in tumor treatment as it limits patients’ treatment options and contributes to tumor recurrence. For example, high infiltration of CD68+ and CD163+ TAMs were associated with a poor response to neoadjuvant chemotherapy in oesophageal cancers (123), and to immunotherapy resistance in bladder cancer (124, 125). Some of the known mechanisms applied by TAMs to mediate chemoresistance are the inhibition of tumor cell death (126), production of cathepsin cysteine proteases (127), and TAMs mediated upregulation of chemoresistance-associated pathways in tumor cells (128). Oxaliplatin resistance, measured by the reduction in tumor cell death and an increase in autophagy, was observed in SMMC-7721 and Huh-7 human HCC cell lines cocultured with PMA-treated THP-1 macrophages (126). In PyMT cells, TAMs derived cathepsin was shown to inhibit paclitaxel mediated tumor cells death (127). Cisplatin resistance has been shown to be mediated by TAMs derived miR-223 exosomes which activated the PTEN-PI3K/AKT pathway in SKOV3 human epithelial ovarian cancer cells (128). In addition to chemoresistance, TAMs have been reported to be involved in radiotherapy resistance *via* activation of monocyte recruitment pathways. Increased TAMs infiltration and a restored protumorigenic TME were observed as a result of radiotherapy induced activation of CCL2/CCR2 axis in pancreatic ductal adenocarcinoma (129) and of the CSF-1/CSF-R axis in glioblastoma (120).

To date there have been several investigations that have reported on TAM phenotypes present in a specific TME and this knowledge is proving invaluable for the understanding the role of TAMs in tumor development and discovery of an effective treatment (130). For example, in breast cancer the following macrophage phenotypes and their functions have been described: (i) M2a TAMs mediated the persistence of the dormant breast cancer cells (131), (ii) M2a/M2c (CD163+) TAM infiltration was associated with poor differentiation, fast proliferation, histological ductal type, and estrogen receptor negativity in primary breast cancer (132); (iii) CCR6 expressing TAMs were involved in the initiation and early stage tumorigenesis of breast cancer *in vivo* (133), (iv) high infiltration of SIGLEC1+ CCL8+ TAMs was shown to be associated with shorter disease-specific survival in estrogen receptor positive breast cancer (134), (v) high infiltration of CCR5-expressing macrophages was found in residual epidermal growth factor receptor 2 (Her2) positive breast tumors (73).

In a lung cancer (A549) xenograft model M0, M2a and M2c TAMs were found to promote cancer invasiveness, while M1 macrophages contributed to tumor suppression, reduced angiogenesis and sensitivity to chemotherapy (29). M2a TAMs were found to also be involved in A549 tumor cell migration *via* direct contact with tumor cells (135).

In HCC, a high infiltration of CD86low/CD206high (defined as M2) TAMs correlated with increased tumor aggressiveness, poor overall survival (OS) and increased tumor time to recurrence (TTR) in α-fetoprotein-negative patients. Conversely, a high number of CD86high/CD206low (M1 defined) TAMs was associated with favorable OS and TTR (136). M2c TAMs were seen in early stages of HCC in C57BL/6 male mice on high fat–high cholesterol–high sugar diet treated with a hepatocarcinogen (diethyl nitrosamine) (137). M2b (defined as CD14+CD68+CCL1+IL-12-IL-10+iNOS-) macrophages were also found as a dominant population in advanced HCC (31).

Immunohistochemical analysis of OSCC showed significant TAMs infiltration compared to healthy tissues (138) and the presence of CD68+CD163+(M2) TAMs or CD206+ (M2-like) TAMs were associated with poor overall survival (139, 140). In addition, CD163+ (M2) TAMs were associated with primary HPV-negative HNSCC (119).

Overall, data accumulated through several different cancer models suggest that TAMs are a heterogeneous group of cells with multiple mechanisms involved in supporting of tumor progression. Studies indicate that increased infiltration of M1 macrophages, that are pro-inflammatory and equipped with many different cytotoxic molecules important in suppressing tumorigenesis. Alternatively, the predominance of alternative macrophage phenotypes, of any M2 subtype, compromises tumor suppression. Indeed, tumorigenesis is a complicated process with TME being variable between different tumor types and/or tumor stages. And that is why studies looking at polarized macrophage phenotypes report variety of TAMs phenotypes. It is clear, however, that more investigations are needed to identify the TAMs phenotypes in specific TME and possible interplay between these populations for the development of effective therapeutic strategies targeting TAMs.

### TAM Targeting Strategies: Strategies in Targeting TAM Recruitment

**Figure 4:**
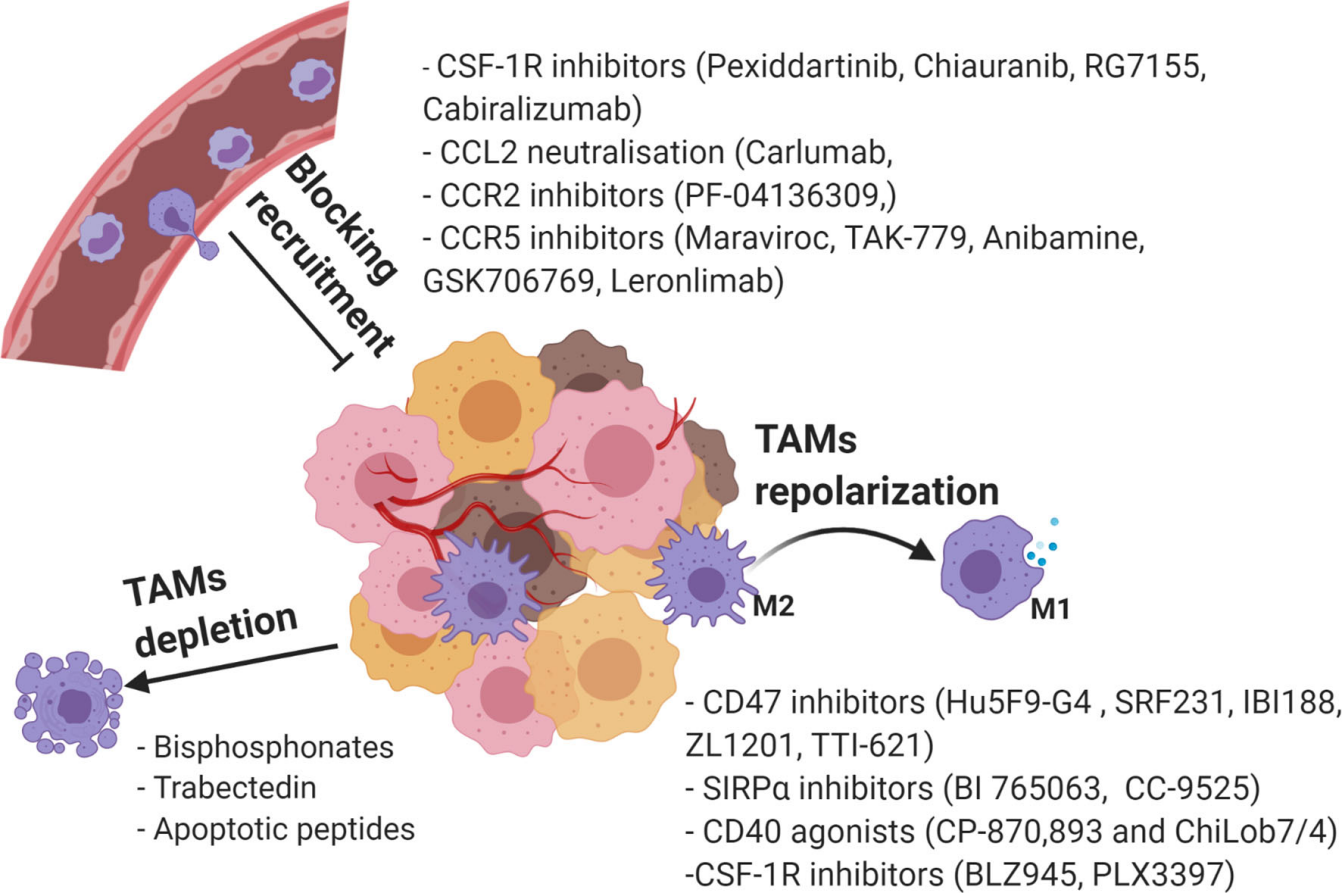
Current strategies in targeting TAMs as a novel cancer therapy. Created with BioRender.com.

Considering the various roles of TAMs in tumorigenesis, significant research effort has focused on targeting of protumorigenic TAMs as potential cancer therapies. Among the widely applied strategies are suppression of TAM recruitment, phenotypic reprograming towards anti-tumorigenic M1, and TAMs depletion (141) (**Figure 4**).

The inhibition of monocyte-derived macrophage recruitment is heralded as a promising strategy to reduce the tumor associated TAMs population and to boost an anti-tumor response. The M-CSF/CSF-1R, CCL2/CCR2, CCL5/CCR5, and CX3CL1/CX3CR1 pathways discussed above are the main targets that show potential in tumor therapy (142).

Several monoclonal antibodies and small molecule inhibitors of CSF-1R are under development as a monotherapy or in combination with other therapeutic agents. The FDA approved small molecule inhibitor of CSF-1R Pexidartinib (PLX-3397), used for the treatment of tenosynovial giant cell tumor (143), has been further investigated in multiple clinical trials for treatment of other solid tumors. The recent phase Ib clinical trial of PLX-3397 in combination with paclitaxel (a chemotherapeutic) in patients with advanced solid tumors reported effective blockage of CSF-1R measured by the decline in peripheral blood monocytes and increase of CSF1 levels in plasma. Complete response and partial response were seen in 3%(n=1) and 13% (n=5) of patients, respectively. However, PLX-3397 does have side effects; 70% of patients (n=38) reported Grade 3-4 adverse effects, which were hematological toxicities (anemia and neutropenia) and non hematological (hepatotoxicity due to possible effect on Kupffer cells and hypertension) (144).

Chiauranib (CS2164), another small molecule targeting CSF-1R and angiogenesis-related kinases (VEGFR2, VEGFR1, VEGFR3, PDGFRα and c-Kit), showed significant antitumor activity in several human xenograft models (145). Currently Chiauranib is being investigated in two ongoing clinical trials on solid tumors (www.clinicaltrials.gov) (ClinicalTrials.gov Identifiers: NCT04830813, NCT03974243) and one Phase I completed trial (ClinicalTrials.gov Identifier: NCT02122809) that has confirmed its safety and favourable pharmacokinetics (146).

Several CSF-1R blocking antibodies were also developed and investigated in clinical trials as an anti-cancer therapy. A CSF-1R blocking monoclonal antibody RG7155 was shown to reduce CSF-1R+CD163+ TAMs and peripheral blood CCR2+ monocytes in phase I clinical trial in patients with diffuse-type giant cell tumor (ClinicalTrials.gov identifier NCT01494688) (147). Also, safety and pharmacodynamic activity of CSF1R inhibitor cabiralizumab was reported in phase I clinical trial testing a combination of CD40 agonist APX005M (sotigalimab) and cabiralizumab with or without PD-1/PD-L1 inhibitor nivolumab in PD-1/PD-L1 resistant advanced cancer patients (ClinicalTrials.gov Identifier: NCT03502330) (148).

CSF-1R blocking agents are also being investigated for synergistic effects with current cancer therapeutics in preclinical studies. For instance, an increase in doxorubicin potency was observed in combination with BLZ945 in mice ovarian cancer model (149) and PLX3397 in mice castration-resistant prostate cancer (150). Halbrook et al. also reported that a combination of gemcitabine and CSF-1R inhibitor AZD7507 showed better treatment outcomes *in vivo* (151).

The blocking of the CCL2/CCR2 axis is also under investigation in multiple clinical studies with variable therapeutic outcomes. For instance, Carlumab (CNTO888), an IgG1k monoclonal antibody that binds to CCL2 showed low therapeutic effect with an increase in free CCL2 levels in phase I trials in patients with solid tumors (152) and in a phase 2 study in metastatic castration-resistant prostate cancer (153). On the other hand, targeting the CCR2 receptor was shown to be more promising, as the combination of a CCR2 inhibitor (PF-04136309) with FOLFIRINOX (oxaliplatin and irinotecan plus leucovorin and fluorouracil) improved clinical outcomes in patients with borderline respectable and locally advanced pancreatic adenocarcinoma in a phase I clinical trial (Clinical Trials.gov Identifier: NCT01413022). In the PF-04136309 with FOLFIRINOX combined treatment group, control of tumor growth was seen in 32 out of 33 patients with significant reduction in tumor markers. In addition, there was a decrease of circulatory CCR2+ monocytes, an increase of bone marrow CCR2+ monocytes, a reduction of TAMs in tumor tissues and modulation of the TME (increase in IL-12 and TGF-α, and a decrease in IL-10, TGF-b, IL-13) (154). In metastatic PDAC, the combination of PF-04136309 with nab-paclitaxel and gemcitabine was shown to have an increase pulmonary toxicity in Phase Ib (Clinical Trials.gov Identifier: NCT02732938) (155). Though, the CCL2/CCR2 axis is an important target for exploration, Kitamura et al. showed that blocking CCL2/CCR2 axis downstream molecules could be more efficient. The study found that CCL2 recruited metastasis-associated macrophages (MEMs) which accumulated in metastatic sites *via* activation of the CCL3/CCR1 axis. The suppression of the CCL3/CCR1 axis reduced MEMs accumulation with no effect on circulating monocytes, resident macrophages and CD11b+ lung macrophages numbers, indicating that targeting the CCL3/CCR1 axis may have high treatment specificity (69).

Targeting the CCL5/CCR5 axis has been shown to be a potent strategy to limit TAM recruitment and modulate TAM phenotype. For example, antitumor responses were observed after treatment with CCR5 and CCL1 inhibitors such as CCL5 and CCR5 blocking antibodies, and CCR5 antagonists (Maraviroc, TAK-779, Anibamine, and GSK706769) (156, 157). Blocking the CCR5 receptor with either Maraviroc, CCL5 neutralizing antibody, or CCLR5 blocking antibody was investigated in human colorectal cancer explant model. This study found that inhibition of the CCL5/CCR5 axis induced M1-like TAMs polarization, which mediated antitumor responses. Further investigation of Maraviroc in a pilot clinical trial (MARACON) (ClinicalTrials.gov identifier: NCT01736813) in patients with advanced metastatic colorectal cancer showed therapeutic efficiency with a tumor control rate of 80% (4 out of 5 patients had partial response or stable disease) and mild side effects (158). In a preclinical study, Maraviroc administration lowered F4/80+ TAM numbers and decreased tumor growth in metastatic BM1 MDA-MB-231 human triple negative breast cancer (74). Among CCR5 blocking antibodies, a humanized anti-CCR5 Leronlimab in combination with Carboplatin is currently under investigation in phase 1b/2 clinical trial ClinicalTrials.gov identifier: NCT03838367) for patients with CCR5+ metastatic triple negative breast cancer (159).

Another potential target to prevent macrophage recruitment is to block the CX3CL1/CX3CR1 pathway. Though several CX3CL1/CX3CR1 blocking agents have been developed (160–162) and anti-tumor activity was reported for CX3CR1 receptor agonists JMS-17-2 and KAND567 (162, 163), the effect of CX3CL1/CX3CR1 pathway inhibition on TAMs has not been explored.

Overall, targeting TAMs recruitment with small molecules or antibodies has been shown to be a promising treatment technique that can be used as a monotherapy or combination therapy with standard treatment therapeutics. However, given the need to address the side effects in targeting macrophage recruitment other strategies for targeting TAMs are under investigation.

### TAM Targeting Strategies: Strategies to Re-Polarize TAMs Towards an Anti-Tumorigenic Phenotype

Macrophage plasticity can also be used as an opportunity to treat cancer by repolarizing TAMs to become anti-tumorigenic. This strategy has already been shown to be possible as the FDA-approved cancer chemotherapy paclitaxel and sorafenib can repolarize TAMs (164, 165).

Preclinical and clinical data indicates that enhancement of phagocytosis by TAMs promotes tumor cell clearance and antigen presentation (166). Tumor cells evade macrophage phagocytosis by expressing CD47 that sends “do not eat me signal” to phagocytic cells *via* interaction with macrophage signal regulatory protein alpha (SIRPα) receptor. CD47 was found to be present in numerous haematological and solid tumor types and its expression was associated with reduced survival (167). For that reason, blocking the CD47-SIRPα axis has been widely investigated in cancer treatment with the reported treatment effects being enhancement of tumor cell phagocytosis, TAM repolarization towards an M1 phenotype, increased DC mediated antigen presentation to CD4 and CD8 T cells, enhancement of NK-mediated Antibody Dependent Cellular Cytotoxicity and caspase-independent tumor cells apoptosis (168). Currently, there are few anti-CD47 antibody therapeutics, anti-SIRPα antibody therapeutics or recombinant SIRPα protein in preclinical and clinical studies. The clinical results of CD47-SIRPα disruption was evaluated in Phase I trial of Hu5F9-G4 (5F9) (ClinicalTrials.gov identifier: NCT02216409), a humanized IgG4 anti-CD47 antibody in patients with advanced solid tumors and lymphomas. Treatment was well tolerated and antitumor activity was observed in two patients with clear cell ovarian and fallopian tube carcinomas. However, a few adverse effects were observed in the initial stages of treatment, such as transient anemia, observed in 57% of patients after the initial priming dose. This was an anticipated side effect, because CD47 is expressed on erythrocytes (RBCs). Stabilization of hemoglobin levels was maintained by rapidly released young RBCs which are resistant to 5F9 due to the lack of CD47 on their surface (169). Preliminary data of the Phase 1/1b clinical trial (Clinical Trials.gov Identifier: NCT03512340) reported the safety and tolerability of SRF231, a fully human IgG4 anti-CD47 antibody, in patients with advanced solid and hematological tumors. Unfortunately, complete or partial response from treatment has not been observed, but, participants had prolonged stable disease (170). Three more anti-CD47 antibodies, IBI188 (ClinicalTrials.gov Identifier: NCT03763149 and NCT03717103), ZL1201 (ClinicalTrials.gov Identifier: NCT04257617), TTI-621 (ClinicalTrials.gov Identifier: NCT02663518, NCT02890368) are in Phase I clinical trials (www.clinicaltrial.gov) with no reported results as of the time of publishing.

Two other, monoclonal antibodies directed against the CD47 receptor SIRPα (BI 765063 and CC-95251) are both under Phase I investigations (ClinicalTrials.gov Identifier: NCT03990233, ClinicalTrials.gov Identifier: NCT03783403 respectively). Though no clinical data is available regarding targeting SIRPα with antibodies, a possible neurological side effect could be anticipated as SIRPα is expressed on neurons (171). The efficiency of SIRPα fusion proteins is also under investigation in Phase I clinical trials: TTI-621 (ClinicalTrials.gov Identifier: NCT02663518) and ALX148 (Clinical Trials.gov Identifier: NCT03013218) as a monotherapy or in combination with other treatment agents.

Other methods to enhance TAM antitumor activity are also under investigation. For example, the effectiveness of antigen presentation to effector lymphocytes is strongly dependent on CD40/CD40L binding. CD40 is a receptor present in antigen presenting cells (APCs) including macrophages and its ligand, CD40L is expressed on CD4 T cells, B cells and NK cells and memory CD8 T cells. Stimulation of CD40 receptor on APCs was shown to enhance antitumor immunity in preclinical and clinical studies (172–175). Several approaches to activate CD40/CD40L are under investigation: targeting CD40 by recombinant agonistic CD40 antibodies or targeting CD40L by using recombinant CD40L or adenovirus vectors carrying CD40L gene (172). Among the most studied CD40 agonists are CP-870,893 and ChiLob7/4. The CD40 agonist CP-870,893, a fully human monoclonal antibody in combination with gemcitabine, was shown to be well tolerated and induced the development of antitumor immunity in patients with advanced pancreatic cancer (Clinical Trials.gov Identifier: NCT00711191) (173). Antitumor immunity was also induced when the CD40 agonist CP-870,893 was used in combination with carboplatin and paclitaxel in advanced melanoma and other solid tumors patients, such as hormone-independent prostate cancer, renal cell carcinoma and ovarian cancer patients (174). A phase I clinical trial using the IgG1 chimeric anti-CD40 antibody ChiLob7/4 found it to be well tolerated in patients with various tumors and 15/29 patients showed median 6 months disease stability (ClinicalTrials.gov Identifier: NCT01561911) (175).

Another promising strategy to modulate protumorigenic TAMs is to inhibit CSF-1R. Though the CSF-1/CSF-1R pathway is required for TAM recruitment and survival, the inhibition of CSF-1R by BLZ945 was shown to repolarize TAMs in a glioblastoma multiforme mouse model. In addition to the reduction in M2 associated gene expression (*Adm, Arg, Mrc1, F13a1*) in TAMs, this study observed multiple therapeutic effects from BLZ945 treatment, such as reduced tumor aggressiveness and increased animal survival. The effects on TAM survival and polarization were found to be mediated by tumor derived factors, these being IFNγ and GM-CSF (176). TAM repolarization and consequent tumor suppression were also observed after treatment with another CSF-1R inhibitor, PLX3397, in a mouse glioblastoma model (177). Interestingly, both studies reported that treatment with CSF-1R inhibitors did not reduce TAM numbers in the TME, however, microglia depletion was seen in the surrounding brain tissues (176, 177). Antitumor activity and modulation of TAMs phenotype were observed after PLX3397 administration in a hepatocellular carcinoma mouse model, where TAM repolarization was mediated by tumor derived CSF-2 (178). Collectively, these studies indicate the potential of CSF-1R targeting to block M2-like TAM polarization, however, off-target effect such as the suppression of macrophages in healthy tissues may be a side effect to be investigated.

Other promising treatment targets include; M2 specific scavenger receptor MARCO, which was associated with poor prognosis in breast cancer, metastatic melanoma and NSCLC (179); PI3Kγ signalling that mediates immunosuppressive reprograming of macrophages (180); Lrg4/Rspo-1 axis that was shown to support M2 polarization (181); and tumor-derived exosomes that have been shown to support M2 TAMs polarization and metastasis (182, 183).

Overall, repolarization of macrophages is showing promise as a new therapeutic strategy in cancer treatment. Though clinical data on the treatment efficiency of repolarizing agents is limited, it is clear that CD47-SIRPα, CD40/CD40L, and CSF-1/CSF-1R molecular pathways are involved in tumor survival and progression. Thus, antitumor immunity can potentially be boosted by reprogramming M2 macrophages to more M1-like phagocytic cells able to enhance tumor immunologic clearance.

### TAM Targeting Strategies: Strategies in Depleting TAMs in the Tumor Microenvironment

Several chemotherapeutic agents also have been reported to be cytotoxic to TAMs. Bisphosphonates, used in treatment of bone metastatic cancers like breast cancer and prostate cancer (184), also have a macrophage-killing properties in addition to killing tumor cells (185). Among the most used bisphosphonates are clodronate and zoledronic acid which are administered as a single agent or in combination with others to target multiple tumorigenic components. As a drug, bisphosphonates have low bioavailability (short plasma half-life, rapid uptake within bone and rapid kidney excretion) (186) and cause severe side effects (jaw osteonecrosis, atrial fibrillation, nephrotoxicity, stress fractures and gastrointestinal lesions) (187) that limit their use and therapeutic potential. Thus, bisphosphonates nanodelivery has more advantages over conventional drug administration. Clodronate liposomes suppresses tumor growth and angiogenesis (22, 23) and zoledronic acid in the form of lipid-coated calcium zoledronate nanoparticles also depletes TAMs and attenuates tumor growth (188, 189).

Trabectedin is a chemotherapeutic agent used for treatment of advanced soft tissue sarcomas and recurrent ovarian cancer. Apart from having a cytotoxic effect on cancer cells, trabectedin also induces caspase 8-mediated apoptosis in monocytic phagocytes (macrophages and monocytes) *via* activation of TNF-related apoptosis-inducing ligand (TRAIL) receptors signalling (190). Trabectedin mediated TAM depletion and the reduction in tumor volume was observed in trabectedin-resistant murine MN/MCA1 fibrosarcoma and a xenograft model of ovarian cancer (IGROV). In addition, immunohistochemistry of tumor biopsies from cancer patients receiving trabectedin, showed a reduction of TAMs infiltration and blood vessels density compared to biopsies collected before chemotherapy initiation (190). A structurally related compound to trabectedin, lurbinectedin, also has macrophage-depleting properties. *In vivo* lurbinectedin administration induced TAM depletion in xenografted PDAC (191), xenograft human ovarian cancer and mouse fibrosarcoma MN/MCA1 models (192). In addition, treatment synergy in reducing tumors was found when lurbinectedin was administered in combination with gemcitabine (191). This treatment combination was also well tolerated in Phase I clinical trials in patients with advanced solid tumors (Clinical Trials.gov Identifier: NCT01970553) (193).

Overall, the above studies demonstrate that TAM depletion has tremendous potential as a novel cancer treatment as a monotherapy but also in combination with traditional therapies. However, many of these treatments, in addition to severe side effects (186, 187, 194), will reduce the systemic macrophage populations, which are a first line of defense in the innate immune response. Considering the importance of macrophages in initiating the immune response, nanotargeting of TAMs has a potential for the development of more specific treatment with a potential low effect on the systemic macrophage population.

### Peptides as a Strategy to Targeted TAMs

Nanomedicine provides a novel approach in cancer treatment. Compared to traditional chemotherapeutic treatment, specific targeting of TAMs, rather than macrophages more broadly, has the potential to increase treatment potency and reduce side effects of treatment. Several TAM specific peptides are currently being investigated and these include M2pep (195), “UNO” (196), melittin (197), RP-182 (198), IL4RPep-1 (199), T4 peptide (200), Pep-20 (201), and CRV (202) (**Table 2**).

**Table 2:** TAMs targeting peptides.

Peptide	Sequence	Target	Function	References
M2pep	YEQDPWGVKWWY-OH	Murine CD45+F4/80+CD301+TAMs	Targeting	(195)
Cyclic M2pep(RY)	CGYEQDPWGVRYWYGC-OH	Murine CD45+F4/80+CD301+TAMs	Targeting	(203, 204)
Melittin	GIGAVLKVLTTGLPALISWIKRKRQQ-NH2	CD206 TAMs	Targeting	(197)
“UNO” peptide	CSPGAKVRC-OH	CD206 TAMs	Targeting	(196)
RP-182 peptide	KFRKAFKRFF-OH	CD206 TAMs	Targeting; M2 macrophages apoptosis; M2 macrophages repolarization towards M1	(198)
T4	NLLMAAS-OH	Tie2+ TAMs and endothelial cells.	Targeting; Blocks Tie2/Ang1	(205)
IL4RPep-1	CRKRLDRNC-OH	IL-4R+ TAMs and tumor cells.	Targeting	(199)
Pep-20	AWSATWSNYWRH-NH2	CD47	Targeting; Block CD47/SIRPα	(201)
Pep-20-D12	a w s ATWSNY w r h-NH2*	CD47	Targeting; Block CD47/SIRPα	(201)
CRV	CRVLRSGSC-OH	TAMs retinoid X receptor beta	Targeting	(202)

*Lower case letters represent D-amino acids.

Cieslewicz et al. used phage peptide display libraries PhD C7 and PhD12 to identify a peptide called M2pep that was found to bind to murine TAMs (CD45+F4/80+CD301+) *in vitro* and *in vivo*. M2pep successfully delivered a proapoptotic peptide to CT-26 colon cancer cells, which led to the reduction of TAM populations and improved survival in a murine model (195). Currently M2pep is an actively researched macrophage targeting peptide that was used to develop various nanocarriers to deliver CSF-1/CSF-1R inhibitors like PLX3397 and CSF-1R-siRNA (206, 207), and a proapoptotic peptide (KLA peptide) (195). Several studies also have addressed the enhancement of M2pep targeting and stability. For example, M2pep(RY) with amino acid substitutions in M2pep (lysine-9 to an arginine, K9R and tryptophan-10 to a tyrosine, W10Y) and decafluorobiphenyl cyclisation through cysteines significantly enhanced binding affinity and increased serum stability (203, 204). Another study modified M2pep(RY) by tyrosine substitution with 3,5-diiodotyrosine to develop a pH sensitive M2pep(RY). This peptide showed increased selectivity to IL-4 polarized (M2) macrophage over IFN-γ and LPS polarized (M1) macrophages at pH 6 compared to unmodified M2pep(RY) peptide *in vitro* (208). Despite significant research into M2pep delivery of anti-cancer drugs and optimization of targeting, the macrophage molecule that M2pep binds to remains unknown.

The ability of certain peptides to specifically bind to CD206+ (M2) macrophages was demonstrated for melittin, RP-182 peptide and “UNO” peptide (196–198). Melittin, a major component of honeybee (Apis mellifera L.) venom, is known for its haemolytic and cytotoxic properties. Lee et al. found that a non-cytotoxic dose of melittin displayed CD206+ (M2) TAM selectivity, without inhibiting the CD86+ (M1) macrophage population or other leukocytes (197). These findings were supported by an *in vivo* investigation, where mice inoculated with mouse Lewis lung carcinoma and then treated with melittin and proapoptotic peptide (KLAKLA)_2_ had reduced CD206+ (M2) TAM tumor infiltration, reduced tumor growth, and angiogenesis compared to control mice (197).

Peptide RP-182 was discovered by *in silico* screening of host defense peptides. RP-182 was found to bind to human and murine CD206+ (M2) TAMs and induce apoptosis and/or repolarization towards a proinflammatory anti-cancer CD86+ M1-like phenotype (198). The *in silico* modelling predicted that RP-182 may also bind to the receptors transglutaminase 2 (TGM2), RelB (a members of the NF-κB family), SIRPα, and CD47 (209).

“UNO” peptide was identified by *in vivo* phage display and its GSPGAK motif (also termed as “mUNO” peptide) is found in several CD206 physiological ligands. Though the “UNO” peptide is cyclic, binding to CD206 requires peptide linearity which is enabled by the reducing conditions found in the TME. The “UNO” peptide was also able to bind to and be internalized by CD206+ expressing (M2) murine TAMs and human CD206+ (M2) macrophages (196, 210, 211). In five independent *in vivo* solid tumor models, a fluorescently labelled “UNO” peptide (FAM-UNO) was found to accumulate in tumor tissues infiltrated with CD206+ (M2) TAMs, but not in non-malignant adjacent tissues or in controlled animal tissues. In addition to intra-tumoral TAM homing, FAM-UNO has also the ability to accumulate in sentinel lymph nodes, which was observed in *ex vivo* AT1 tumor model (196). The ability of “UNO” peptide to deliver a therapeutic cargo was demonstrated by the increased accumulation of UNO conjugated paclitaxel-loaded polymersome to CD206+ (M2) TAMs in MCF-7 breast cancer bearing mice (196). In addition, “mUNO” peptide conjugated with toll like receptor agonist TLR7/8 (resiquimod)-loaded lignin nanoparticles was shown to target CD206+ (M2) TAMs *in vivo* in an aggressive mice triple-negative breast cancer model (212). All these findings suggested that the “UNO” peptide is a good candidate for the development of highly specific cancer therapies and also can be used in imaging (196, 212).

Tyrosine-protein kinase receptor (Tie2) expressing monocytes and macrophages (TEMs) are involved in tumor angiogenesis and contribute to tumor aggressiveness. Besides TEMs, Tie2 receptor is found on endothelial cells and upon interaction with Ang1 and Ang2 this pathway has a substantial role in the formation of new vasculature (213, 214). Targeting tumor Tie2-mediated angiogenesis is a potential therapeutic strategy to prevent tumor access to nutrients and oxygen. The T4 peptide (NLLMAAS) was found to bind to human recombinant Tie2 receptor and was identified by a phage display peptide library testing (205). The study reported that T4 peptide blocked Tie2 interaction with Ang1 in a HUVEC cell line *in vitro* and inhibited angiogenesis observed *in vivo* in a chick chorioallantoic membrane (CAM) assay (205). T4 was explored for targeting tumor endothelial cells and TAMs to prevent breast cancer relapse in a 4T1 breast cancer cell mouse model (200). For this purpose, T4 peptide was protected from proteolytic degradation by creating a dual-responsive (pH sensitive and enzyme cleavable) (mPEG1000-K (DEAP)-AAN-NLLMAAS) nanoformulation which upon exposure to the TME would expose active T4 peptide. This T4 nanoformulation was able to suppress angiogenesis, delay tumor relapse and metastasis formation in the animal model (200). Two other peptides; GA5 peptide (NSLSNASEFRAPY) and T7 (HHHRHSF) were also reported to bind the Tie2 receptor, although their binding to TEMs requires investigation (205, 215).

The peptide IL4RPep-1 (CRKRLDRNC) has been shown to bind to IL-4R-expressing tumor cells and M2(IL-4) polarized macrophages (199). Vadevoo et al. (199) reported that IL4RPep-1 was able to deliver proapoptotic peptide (KLAKLAK)2 with paclitaxel to a 4T1 breast cancer tumor and this treatment resulted in suppression of tumor growth and metastatic spread in the mouse model. Due to the high expression of IL-4 receptor in M2 macrophages, the number of M2 macrophages was significantly reduced after treatment. In addition, the reduction in Treg cells was observed together with the increase in activated cytotoxic CD8+ T cells in treated mice (199).

Wang et al. (201) discovered that Pep-20 peptide (AWSATWSNYWRH) was able to block CD47/SIRPα pathway by binding to CD47. An *in vitro* study confirmed the ability of Pep-20 to enhance macrophage phagocytic activity against a range of murine and human cancer cell lines as well as promoting macrophage-mediated development of antitumor CD8+ T cells (201). Antitumor activity of Pep-20 was also observed in a CT26 tumor mouse model, where the Pep-20 treated group showed tumor suppression and increased overall survival. In further studies Pep-20 was modified by replacing certain L-amino acid residues with their D-amino acid resides (**Table 1**) in an attempt to make the peptide more proteolytically stable, termed Pep-20-D12. These modifications not only improved serum stability but also increased peptide’s anti-tumor activity against MC38 tumor *in vivo* (201).

Tang et al. (202) reported that cyclic CRV peptide (CRVLRSGSC) had specific binding to TAMs *via* retinoid X receptor beta, a receptor found to be expressed predominantly by TAMs (CD11b+, F4/80+, CD68+). The group also demonstrated peptide specific tumor homing, as fluorescently labelled (sulforhodamine 101) CRV peptide conjugated with porous silicon nanoparticles increasingly accumulated in 4T1 tumor mice model comparing to nanoparticles without CRV peptide (202). CRV peptide was also used to design an immunostimulatory tandem peptide nanocomplexes conjugated with TLR9 ligand ODN1826. This nanoformulation in combination with anti-CTLA4 was tested *in vivo* in B16F10 tumor mice model, where the reduction in tumor volume was observed comparing to anti-CTLA4 combinations with untargeted nanocomplexes or with naked ODN1826 (216).

The above studies demonstrate the exciting potential of TAM targeting peptides to enhance cancer treatment by specific delivery of a therapeutic cargo directly to the tumor site. It is clear that more research is required to evaluate safety and efficiency, but some preclinical data indicate that formulations with TAM targeting peptides potentially can outperform traditional treatments (199, 207). The benefits of using TAM targeting peptides are tumor homing which enables specific targeting and intrinsic therapeutic activity observed in few peptides which can be used as a monotherapy or synergy with the carried cargo.

### Nanomaterials as an Effective TAMs Repolarization Strategy

**Figure 5:**
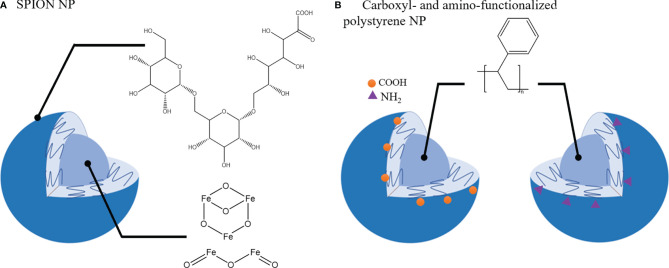
Nanoparticles that induce TAMs phenotypic shift towards tumor-suppressive phenotype. **(A)**. SPION nanoparticle (NP): Carboxy-dextran coating and Iron oxide core. **(B)**. Carboxyl- and amino- functionalized NP with polystyrene core.

A primary function of macrophages is their ability to phagocytose micro- and nano-materials and this process is being exploited to alter and/or modulate macrophage phenotypes. Apart from therapeutic agents to repolarize TAMs towards M1 phenotype, some nanomaterials have been reported to have an intrinsic activity to induce phenotypical changes in macrophages. For example, Zanganeh et al. (217) demonstrated that ferumoxytol, a drug used in treatment of iron deficiency anemia, induced the upregulation of CD86 and TNF-α (M1 macrophage) markers in RAW264.7 macrophages co-cultured with MMTV-PyMT cancer cells. *In vivo* studies confirmed these findings and treatment with ferumoxytol nanoparticles (carboxy-dextran coated super paramagnetic iron-oxide nanoparticles (SPIONs) (**Figure 5A**) induced macrophage mediated suppression of primary tumor growth in MMTV-PyMT cancer model and inhibited metastasis in a KP1-GFP-Luc cancer model (217).

Polystyrene nanoparticles functionalized with carboxyl or amino groups (**Figure 5B**) were also reported to impair CD163 and CD200R expression and IL-10 production in M2 macrophages without affecting the M1 population (218). In the 4T1 tumor model poly(styrene-co-maleic anhydride) (PSMA) nanoparticles conjugated with polymer poly[2-methoxy-5-(2-ethylhexyloxy)-1,4-phenylenevinylene, PPV] were able to attenuate tumor growth and modulate TME by upregulation of M1 macrophage markers (CD86, CD80, iNOS, TNF-alpha) and downregulation of M2-like markers (CD206, CD163) in the TME (219).

Cationic polymers such as cationic dextran and polyethyleneimine (PEI) were shown to alter TAM phenotype *via* TLR4 signalling. The phenotypical changes in TAMs include the upregulation of IL-12, NOS2 and MHCII (M1-related markers) and downregulation of IL-10, Arg1 and Ym1 (M2-specific markers) which were observed *in vitro* and *in vivo* in a S180 sarcoma model. Therapeutically, cationic dextran and PEI showed some anti-tumor activity in tumor bearing wild type mice compared to TLR4 knockout mice (220).

The finding that nanoparticles alone or NPs coated with cationic polymers have immunostimulatory properties that can shift the balance of macrophage phenotypes towards a M1 profile, indicates their exciting potential to enhance therapeutic strategies documented here and contribute to reducing the cancer burden after treatment, particularly when combined with targeting methods.

## Discussion

Macrophages are plastic and highly heterogeneous cells that have a potential to suppress or promote tumor growth. Multiple aspects of macrophage biology are being considered when developing TAM targeting strategies. Many of these strategies are already in clinical studies which gives hope of new effective treatment regimens to be used clinically and also insights into their efficacy. These clinical trials and the research into targeting TAMs has highlighted the porosity in our understanding of the macrophage subtypes present in the TME and their involvement in tumorigenesis. The increase in our understanding of the role of these subtypes in pathology will ultimately aid in the design of potent therapies. An exciting area that is showing a high degree of potential to target TAMs is peptide and nanomaterial targeting due to their ability to modulate the TME without affecting general monocyte/macrophage populations. Indeed, these properties can be used in the development of the future cancer treatment that will be more potent and less toxic.

## Author Contributions

Conceptualization: TH and NO’B-S. Writing and original draft preparation: TH. Writing—review and editing: TH, NO’B-S, JH, WL, JL, and SH. Supervision of TH by NO’B-S, JH, WL, JL, and SH. All authors have read and agreed to the published version of the manuscript. All authors contributed to the article and approved the submitted version.

## Funding

TH was supported by an Australian Government Research Training Program Scholarship. The National Health and Medical Research Council (NHMRC) of Australia and Australian Research Council (ARC) are thanked for financial support over many years for the peptide chemistry and chemical biology studies reported in the authors’ laboratories. NO’B-S is the recipient of NHMRC funding (APP1142472, APP1158841, APP1185426), ARC funding (DP210102781, DP160101312, LE200100163), Cancer Council Victoria funding (APP1163284) and Australian Dental Research Funding in antimicrobial materials and research is supported by the Centre for Oral Health Research in the Basic and Clinical Oral Sciences Division at The Melbourne Dental School.

## Conflict of Interest

The authors declare that the research was conducted in the absence of any commercial or financial relationships that could be construed as a potential conflict of interest.

## Publisher’s Note

All claims expressed in this article are solely those of the authors and do not necessarily represent those of their affiliated organizations, or those of the publisher, the editors and the reviewers. Any product that may be evaluated in this article, or claim that may be made by its manufacturer, is not guaranteed or endorsed by the publisher.
